# Expression and characterization of a β-fructofuranosidase from the parasitic protist *Trichomonas vaginalis*

**DOI:** 10.1186/1471-2091-15-12

**Published:** 2014-06-28

**Authors:** Michael Dirkx, Michael P Boyer, Prajakta Pradhan, Andrew Brittingham, Wayne A Wilson

**Affiliations:** 1 Department of Biochemistry & Nutrition, Des Moines University, Des Moines, IA 50312, USA; 2 Department of Microbiology & Immunology, Des Moines University, Des Moines, IA 50312, USA

**Keywords:** *Trichomonas vaginalis*, Carbohydrate utilization, Invertase, Purification

## Abstract

**Background:**

*Trichomonas vaginalis,* a flagellated protozoan, is the agent responsible for trichomoniasis, the most common nonviral sexually transmitted infection worldwide. A reported 200 million cases are documented each year with far more cases going unreported. However, *T. vaginalis* is disproportionality under studied, especially considering its basic metabolism. It has been reported that *T. vaginalis* does not grow on sucrose. Nevertheless, the *T. vaginalis* genome contains some 11 putative sucrose transporters and a putative β-fructofuranosidase (invertase). Thus, the machinery for both uptake and cleavage of sucrose appears to be present.

**Results:**

We amplified the β-fructofuranosidase from *T. vaginalis* cDNA and cloned it into an *Escherichia coli* expression system. The expressed, purified protein was found to behave similarly to other known β-fructofuranosidases. The enzyme exhibited maximum activity at pH close to 5.0, with activity falling off rapidly at increased or decreased pH. It had a similar K_m_ and V_max_ to previously characterized enzymes using sucrose as a substrate, was also active towards raffinose, but had no detectable activity towards inulin.

**Conclusions:**

*T. vaginalis* has the coding capacity to produce an active β-fructofuranosidase capable of hydrolyzing di- and trisaccharides containing a terminal, non-reducing fructose residue. Since we cloned this enzyme from cDNA, we know that the gene in question is transcribed. Furthermore, we could detect β-fructofuranosidase activity in *T. vaginalis* cell lysates. Therefore, the inability of the organism to utilize sucrose as a carbon source cannot be explained by an inability to degrade sucrose.

## Background

*Trichomonas vaginalis* is the causative agent of trichomoniasis, the most common non-viral sexually transmitted infection worldwide, with over 3 million cases estimated per year in the United States alone [1]. In general, infected men are asymptomatic [2]. However, in women, symptoms of infection may include vaginal discomfort, pain during intercourse and urination, and a thick, yellow, purulent discharge [2]. In most instances, *T. vaginalis* infection is readily treated with the nitroimidazole drugs metronidazole or tinidazole [3]. Likely due in part to the ease with which *T. vaginalis* infections can be treated the disease has been viewed historically as a ‘nuisance’ infection. However, it is now appreciated that infection with *T. vaginalis* predisposes patients to infection with HIV and other sexually transmitted diseases and may also be associated with infertility and adverse pregnancy outcomes [4,5].

Despite decades of study, there are basic questions regarding the biochemistry of this important organism that remain unanswered. We are particularly interested in the means employed by *T. vaginalis* to secure essential nutrients from the environment and are addressing this question through *in vitro* culture of the protist.

As is the case with many parasitic protists, *T. vaginalis* lacks the capacity to synthesize many key metabolites such as saturated/unsaturated fatty acids and purines and pyrimidines [6]. Cultivation of the organism therefore requires use of a complex growth medium [7,8]. The preferred energy source is carbohydrate, although energy-yielding pathways utilizing a variety of amino acids have been identified [6,9,10].

Early work with axenic cultures of *T. vaginalis* demonstrated that glucose, maltose, and glucose polymers such as starch and glycogen, were capable of supporting robust growth [11,12]. The glucose-containing disaccharides lactose and sucrose could not support growth, nor could the glucose-containing trisaccharide raffinose [11,12]. Data relating to the utilization of other mono- and disaccharides is harder to interpret.

Read reported that trehalose, the α1,1 disaccharide of glucose, and melibiose, the α1,6 disaccharide of galactose and glucose, were utilized for growth [12], contradicting earlier studies by Trussell and Johnson [11]. Of the monosaccharides other than glucose that have been tested (galactose, mannose, fructose, xylose, and arabinose), only galactose has been consistently reported to support growth [11,12].

Growth using maltose as a carbon source has been especially well studied in *T. vaginalis*[13,14]. Work by ter Kuile showed that the organism expresses cell-associated maltase activity that cleaves maltose to yield two molecules of glucose, which are then transported into the cell [14,15]. However, other than two preliminary reports of amylase activity associated with the cells, the means by which other disaccharides and more complex carbohydrates are utilized by the organism remains largely uninvestigated [16,17].

The publication of the *T. vaginalis* G3 strain genome sequence in 2007 has provided a wealth of information to guide biochemical investigation [18]. For example, there are a variety of open reading frames that are proposed to encode α- or β-amylases, which could potentially explain the ability of *T. vaginalis* to grow using glycogen or starch [18,19]. Intriguingly, one open reading frame, TVAG_254130, was annotated as a putative β-fructofuranosidase [18,19].

The β-fructofuranosidases (EC 3.2.1.26; commonly referred to as invertases) catalyze the removal of fructose residues from the non-reducing ends of fructose-containing oligo- and/or polysaccharides [20]. The preferred substrate for many such enzymes is sucrose, hydrolysis therefore generating an equimolar mixture of glucose and fructose. In addition to this potential β-fructofuranosidase, some 11 putative proteins, whose closest relatives were sucrose transporters found in plants, are encoded by the *T. vaginalis* genome [18,19]. There is evidence from protein mass spectroscopy that at least some of these sucrose transporters are expressed (Mass Spec of T.vaginalis peptides from Richard Hayes, Patricia Johnson laboratory [version: 2009-05-27] accessed via the TrichDB database [19,21]). Thus, while *T. vaginalis* fails to utilize sucrose as a carbon source, it appears that it may encode the enzymes required to do so.

Here, we describe the cloning of TVAG_254130 and the expression and characterization of its protein product. We established that the TVAG_254130 open reading frame is expressed in *T. vaginalis*. We also determined that β-fructofuranosidase activity could be detected in cell lysates of *T. vaginalis*. We went on to express and purify the product of the TVAG_254130 open reading frame from *E. coli* and determined that it encoded a β-fructofuranosidase with robust catalytic activity towards both sucrose and raffinose. Thus, the inability of sucrose to support the growth of *T. vaginalis* is not due to a lack of capacity to degrade this disaccharide.

## Methods

### Growth and maintenance of trichomonads

*Trichomonas vaginalis* G3 (ATCC PRA-98; Taxonomy ID: 412133) and *Pentatrichomonas hominis* Hs-3:NIH (ATCC 30000) were obtained from the American Type Culture Collection (Manassas, VA). Stock cultures were grown in a trypticase-yeast-extract-maltose (TYM) medium modified in our laboratory from a recipe first described by Hollander (see [22,23] for details). Routine culture of *T. vaginalis* was carried out in 25 cm^2^ tissue culture flasks containing 5 to 10 ml of medium whereas *P. hominis* grew best in 100 mm screw top borosilicate glass culture tubes filled with 12 ml of medium. Both organisms were grown at 35°C. Cell growth and viability were determined by counting the number of intact and motile trichomonads in an aliquot using a Neubauer hemocytometer. All stock cultures were inoculated at a density of 1.0 x 10^5^ cells/ml and were passaged every 48-72 hr. Under these growth conditions, the peak cell density averaged 3.6 x 10^6^ cells/ml.

### Synthesis of *T. vaginalis* cDNA and verification of expression of the TVAG_254130 open reading frame

*T. vaginalis* G3 was grown for 40 hr in TYM under our standard culture conditions described above. Total RNA was isolated using the SV Total RNA Isolation System (Promega, Madison, WI), according to the manufacturer’s instructions. The RNA was divided into aliquots and stored frozen at -80°C prior to use. For verification of expression of the TVAG_254130 open reading frame, one aliquot of RNA was treated with the Affinity QPCR cDNA Synthesis Kit (Agilent, Santa Clara, CA) to generate cDNA whilst a second aliquot was treated similarly but with reverse transcriptase omitted from the reaction mixture. Aliquots of the cDNA synthesis reaction and mock reaction lacking reverse transcriptase were then employed as the template in PCR reactions with primers designed to amplify the TVAG_254130 open reading frame, as described below.

### Cloning and expression of the TVAG_254130 open reading frame

PCR primers WW98 (catatgaatttttcctcacgactaaaattccattttgagcc) and WW99 (gtcgacctaaaatagccaagtgctgtaataaaattcg) were designed to amplify the coding sequence of the TVAG_254130 open reading frame. Primer WW98 introduced an Nde I site at the start codon of the open reading frame and primer WW99 introduced a Sal I site immediately 3’ of the stop codon in order to ultimately facilitate cloning into the pET-28a bacterial expression system (EMD Millipore, Billerica, MA). For cloning of TVAG_254130 the template for PCR was cDNA prepared from the total RNA isolated as described above, but in this case cDNA synthesis was conducted using the ImProm-II Reverse Transcription System (Promega). Ex-Taq polymerase (Clontech Laboratories Inc., Mountain View, CA) was used in the PCR procedure to amplify TVAG_254130. The PCR product obtained was cloned into the pCR2.1-TOPO vector using the TOPO TA Cloning Kit (Life Technologies Corp, Carlsbad, CA) and then sequenced. The sequence obtained was identical to that reported by the *T. vaginalis* genome sequencing project. The PCR product was excised from the pCR2.1-TOPO vector with Nde I and Sal I and cloned into Nde I/Sal I cut pET-28a. The choice of cloning sites resulted in the incorporation of vector sequence encoding an affinity purification tag of six consecutive histidine residues into the 5’ end of the TVAG_254130 open reading frame. The N-terminal sequence of the recombinant protein was thus extended by the sequence MGSSHHHHHHSSGLVPRGSH. The pET-28a vector containing TVAG_254130 was transformed into *E. coli* strain BL21(DE3) for expression of recombinant protein.

The transformed BL21(DE3) were grown at 37°C in 500 ml of LB medium supplemented with kanamycin sulphate (50 μg/ml) until an OD_600 nm_ of 0.6 was reached. Protein expression was then induced by the addition of isopropyl-β-D-thiogalactopyranoside to 1 mM, and the cultures were transferred to 30˚C. After 16 h of induction, cells were collected by centrifugation (5,000 x *g*, 10 minutes, 4˚C), washed and then resuspended with homogenization buffer (50 mM Tris-HCl pH 8.0, 50 mM NaCl, 5% v/v glycerol, 0.5 μg/ml leupeptin, 1 μg/ml pepstatin, 1 μg/ml aprotinin, 1 mM benzamidine HCl, 0.1 mM TLCK and 1 mM PMSF). Benzonase nuclease (EMD Millipore) was added to reduce viscosity and cell lysis was achieved by addition of rLysozyme solution (EMD Millipore). The cell suspension was incubated for 20 min at room temperature with gentle rocking. Cell debris was removed by centrifugation at 18,000 x *g* for 15 min at 4°C. The supernatant containing soluble recombinant β-fructofuranosidase was decanted, filtered through a 0.45 μm pore size syringe end filter, and applied to a Bio-Scale Mini Profinity IMAC cartridge attached to a BioLogic LP chromatography system (Bio-Rad, Hercules, CA) equilibrated with column buffer (20 mM Tris-HCl pH 8.0, 300 mM NaCl, 5 mM imidazole). The column was washed with 20 ml of column buffer, followed by 20 ml of column buffer with the imidazole concentration increased to 10 mM. The recombinant β-fructofuranosidase was eluted from the column by washing with 20 ml of column buffer with the imidazole concentration increased to 250 mM. Fractions containing β-fructofuranosidase activity were pooled together and dialyzed against storage buffer (50 mM HEPES pH 7.0, 50 mM NaCl) then concentrated to approximately 5 mg/ml protein using an Amicon Ultra-4 centrifugal filter unit (EMD Millipore). Glycerol was added to a final concentration of 20% (v/v). The purified β-fructofuranosidase was stored frozen at -80°C, where it was stable for at least 8 months.

### Measurement of β-fructofuranosidase activity

β-Fructofuranosidase activity was assayed using a modification of published procedures [24]. In brief, for studies using purified enzyme, the β-fructofuranosidase was diluted with sodium acetate buffer (20 mM sodium acetate, 50 mM NaCl, 0.05% v/v Triton X-100, pH 5.0) to a protein concentration of approximately 6 μg/ml. The diluted enzyme was then combined with sucrose or raffinose that had been dissolved in sodium acetate buffer without Triton X-100 in a total volume of 100 μl. This gave a final enzyme concentration in the reaction of ~ 27 nM. Control reactions contained sucrose or raffinose but no added enzyme. The samples were incubated at 30°C for 15 min and the reactions were then terminated by incubation in a dry block set to 100°C for 3 min. The samples were cooled on ice and the glucose released by hydrolysis of sucrose was measured in a coupled enzyme assay employing hexokinase and glucose-6-phosphate dehydrogenase [25]. Under these conditions and at a substrate concentration of 250 mM, the rate of glucose production was linear with respect to time for at least 60 min. The action of β-fructofuranosidase on raffinose liberates fructose, which was measured using the same coupled enzyme assay as for glucose but with the addition of 3 U of phosphoglucose isomerase (Sigma-Aldrich, St. Louis, MO) per reaction. When examining the ability of *T. vaginalis* and *P. hominis* cells extracts to degrade sucrose, we grew the organisms under our standard conditions for 40 hrs until a density of approximately 2.5 × 10^6^ cells/ml was reached. Cells were then collected from 10 ml of medium by centrifugation and lysed by the addition of 200 μl of ice cold lysis buffer (25 mM HEPES, 50 mM NaCl, pH 7.0) supplemented with 1 μg/ml leupeptin, 1 μg/ml pepstatin, 1 mM benzamidine-HCl, 0.1 mM TLCK, and 1 mM PMSF. Aliquots of cell lysate were then incubated at 30°C for 30 min with or without the addition of exogenous sucrose (250 mM). Control reactions contained 250 mM sucrose but no cell lysate. Following this incubation period, the glucose and fructose present in the samples was determined by coupled enzyme assay as described above.

### Analysis of enzyme kinetic data

To estimate the K_m_ and V_max_ when either sucrose or raffinose was used as substrate, purified β-fructofuranosidase (~27 nM final concentration) was incubated at 30°C in 20 mM sodium acetate buffer pH 5.0 with varying concentrations of substrate, obtained by serial dilution of a concentrated stock solution prepared in the same buffer. For sucrose, the concentrations used ranged from 0.488 mM to 250 mM whereas a range of 0.156 mM to 100 mM was used for raffinose. The initial rate of sucrose or raffinose hydrolysis was determined by enzymatic measurement of the glucose or fructose released, as described above. The kinetic parameters were then estimated by directly fitting the data to the Michaelis-Menten equation using the Enzyme Kinetics module of SigmaPlot 11 (Systat Software Inc., San Jose, CA).

## Results

### Sucrose fails to support the growth of *T. vaginalis* under our standard culture conditions

**Figure 1:**
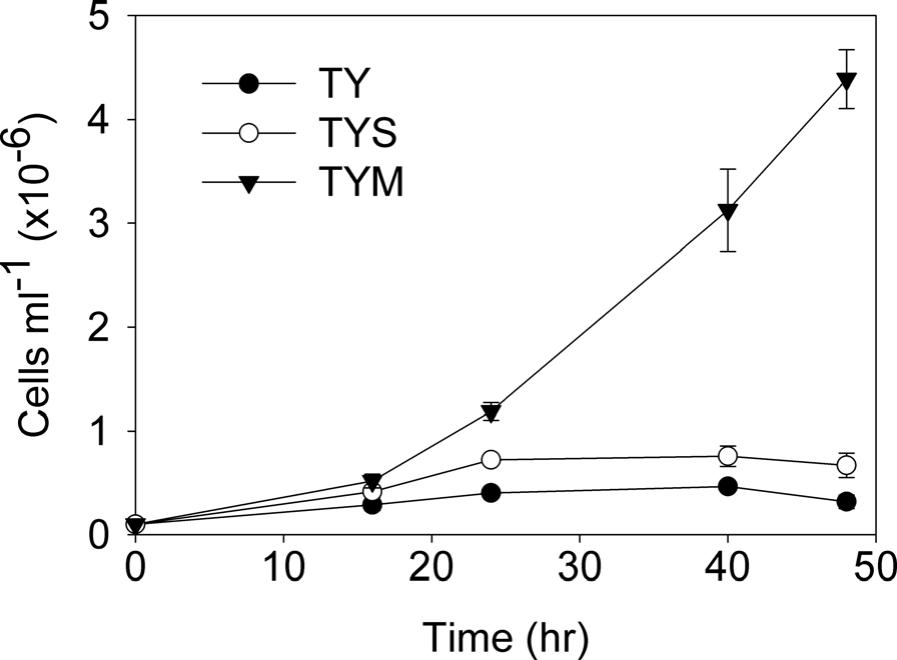
**Sucrose is unable to support the growth of *T. vaginalis*.** TY medium supplemented with maltose (0.5% by weight; TYM), sucrose (0.5% by weight; TYS), or that had no added sugar (TY) was prepared. *T. vaginalis* was grown for 40 hr in TYM. The cells were collected, washed with TY medium and then used to inoculate this same medium, TYM, or TYS at an initial density of 1 x 10^5^ cells/ml. The cell density was then recorded after 16, 24, 40 and 48 hr of growth. Only TYM medium supported robust growth of *T. vaginalis*, with a decrease in motile organisms noted by 48 hr in both TY and TYS media. The results shown are the mean ± standard error for four independent determinations.

The conditions currently used for *in vitro* culture of *T. vaginalis* differ significantly from those used by earlier workers. Thus, we began by determining whether sucrose could fulfil the role of carbon source in our standard TY medium. We prepared TY medium that was supplemented with maltose (0.5% by weight; TYM), sucrose (0.5% by weight; TYS), or that had no added sugar. *T. vaginalis* was grown to a density of approximately 2 × 10^6^ cells/ml. The cells were collected, washed with TY medium without added sugar and then used to inoculate this same medium, TYM, or TYS at an initial density of 1 × 10^5^ cells/ml. The cell density was then recorded after 16, 24, 40, and 48 hr of growth (Figure 1). As expected, the cells grew well in TYM. The cells in both TY and TYS persisted for some 40 hr but there was little increase in cell density in either case. Indeed, there was no statistically significant difference in cell density reached when comparing growth in TY to growth TYS, despite an apparent trend toward higher cell numbers in TYS.

### Cell extracts prepared from *T. vaginalis* contain an activity that hydrolyzes sucrose

Although *T. vaginalis* appeared unable to utilize sucrose as a carbon source, at least under the growth conditions tested, the presence of putative sucrose transporters and a β-fructofuranosidase in the genome prompted us to investigate further. We grew *T. vaginalis* in our standard TYM medium, collected the cells, and prepared lysates. We incubated cell lysates in the presence of sucrose and determined if there was any liberation of glucose and fructose from this added sucrose. As a positive control, we included analysis of *P. hominis*, a trichomonad found in the human gut [26-28], which is able to grow using sucrose as a carbon source ([29], our unpublished observations). We could detect the production of glucose and fructose from sucrose catalyzed by extracts of *T. vaginalis* and *P. hominis* (Table 1). Substantially higher activity was obtained with the samples from *P. hominis* than with the samples from *T. vaginalis*. We were unable to detect any sucrose hydrolysis when using conditioned culture medium in which *T. vaginalis* had been grown for 40 hr as a source of enzyme activity (not shown).

**Table 1:** **Generation of glucose and fructose from exogenous sucrose by extracts of *T. vaginalis***

	**Glucose/fructose formed (nmol/min/mg)**	
	*T. vaginalis*	*P. hominis*
(-) Sucrose	38.5 ± 1.4	52.4 ± 4.6
(+) Sucrose	56.0^a^ ± 2.3	172^a^ ± 13.8

^a^Significantly different from value obtained without added sucrose (p < 0.001, Student’s *t* test).

Extracts were prepared from samples of *T. vaginalis* and *P. hominis* grown and collected as described under ‘Methods’. To determine the ability of cell extracts to degrade sucrose, aliquots of cell extract were incubated with (+) or without (-) 250 mM sucrose and the glucose/fructose produced was measured by coupled enzyme assay. Data are expressed per milligram of soluble trichomonad protein. The results shown are the mean ± standard error for five (*T. vaginalis*) or three (*P. hominis*) independent experiments.

### The open reading frame TVAG_254130, encoding a putative β-fructofuranosidase is expressed in *T. vaginalis* under our standard growth conditions

**Figure 2:**
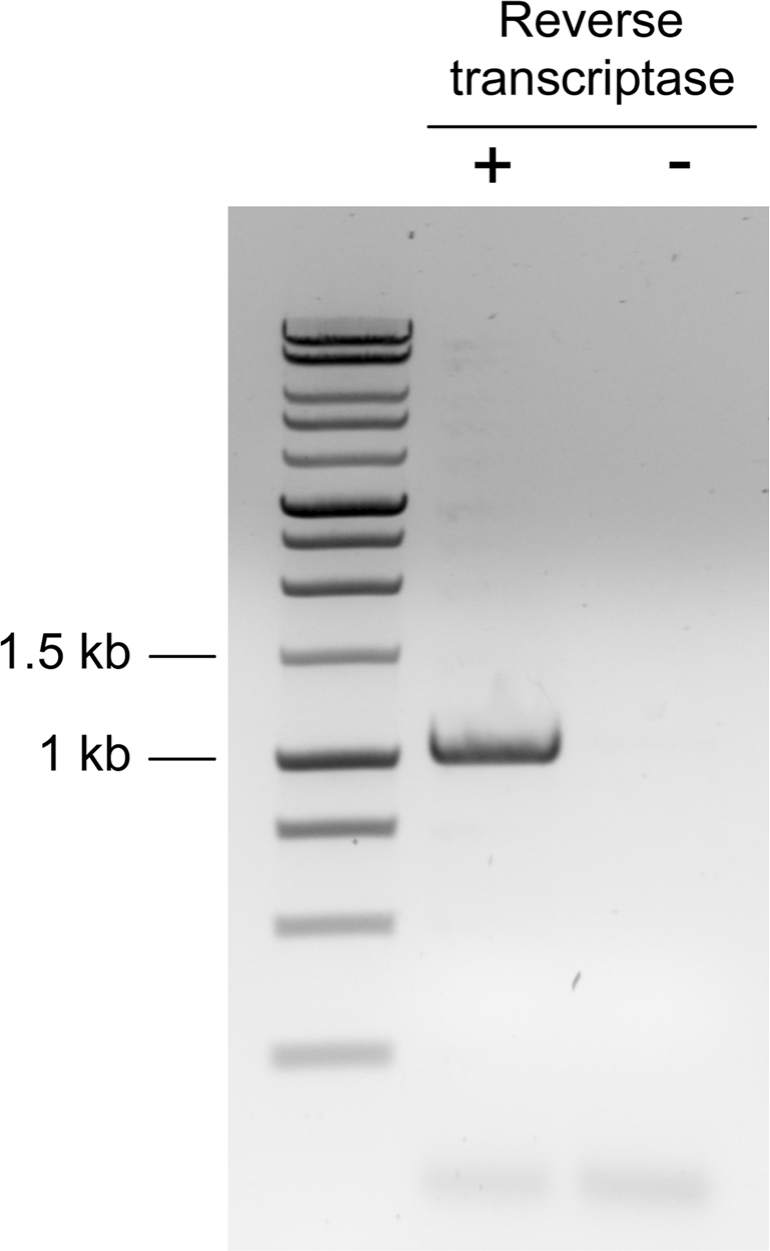
**Amplification of the TVAG_254130 open reading frame from *T. vaginalis *cDNA.** Total RNA was isolated from a 40 hr culture of *T. vaginalis* grown under our standard conditions. The resulting RNA was used to synthesize cDNA (+RT). To control for genomic DNA contamination, mock reactions were carried out in which reverse transcriptase was omitted from the cDNA synthesis step (-RT).

An obvious candidate for the activity responsible for cleavage of sucrose in *T. vaginalis* is the putative β-fructofuranosidase encoded by TVAG_254130. To determine if the protein product of TVAG_254130 could account for the observed sucrose hydrolysis, we first determined if this open reading frame was expressed under our standard growth conditions. Total mRNA was isolated from *T. vaginalis* grown in TYM and cDNA was generated. We were able to amplify the TVAG_254130 open reading frame from cDNA, indicating that it was indeed expressed (Figure 2).

### TVAG_254130 encodes an active β-fructofuranosidase

In order to determine if TVAG_254130 encoded an active β-fructofuranosidase, we cloned the open reading frame into an *E. coli* expression system. Upon induction of expression, we could detect robust hydrolytic activity towards sucrose in extracts transformed with vector containing TVAG_254130 (~10 μmol/min/mg) and there was no detectable activity in extracts from cells transformed with empty expression vector. Thus, TVAG_254130 encodes an active β-fructofuranosidase.

**Figure 3:**
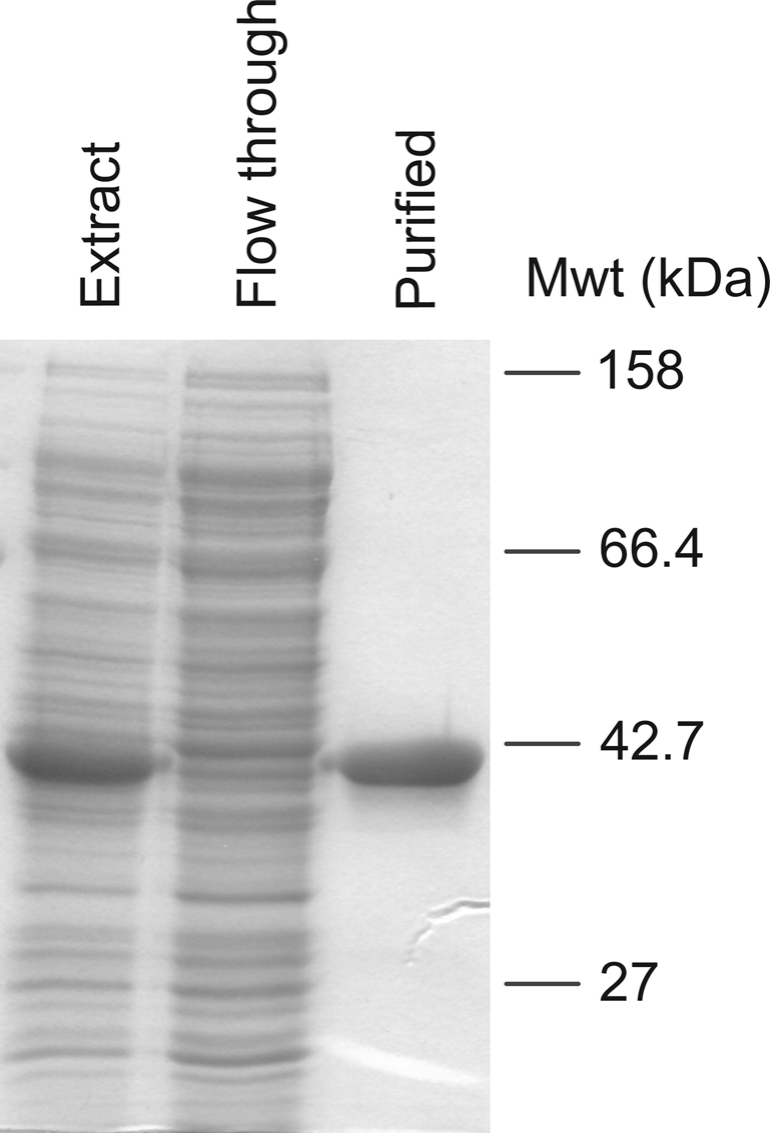
**Analysis of purified recombinant *T. vaginalis *β-fructofuranosidase by polyacrylamide gel electrophoresis under denaturing conditions.** The *T. vaginalis* β-fructofuranosidase encoded by TVAG_254130 was expressed in *E. coli* and purified as described under ‘Methods’. Aliquots corresponding to ~ 25 μg of crude cell extract (*Extract*) or column flow through (*Flow through*) were analyzed along with ~ 5 μg of purified β-fructofuranosidase (*Purified*).

The expression vector chosen introduced a tag of six consecutive histidine residues at the N-terminus, allowing one-step purification by immobilized metal affinity chromatography. The purified protein appeared homogenous by polyacrylamide gel electrophoresis and the yield was approximately 8 mg/L of *E. coli* culture (Figure 3).

**Figure 4:**
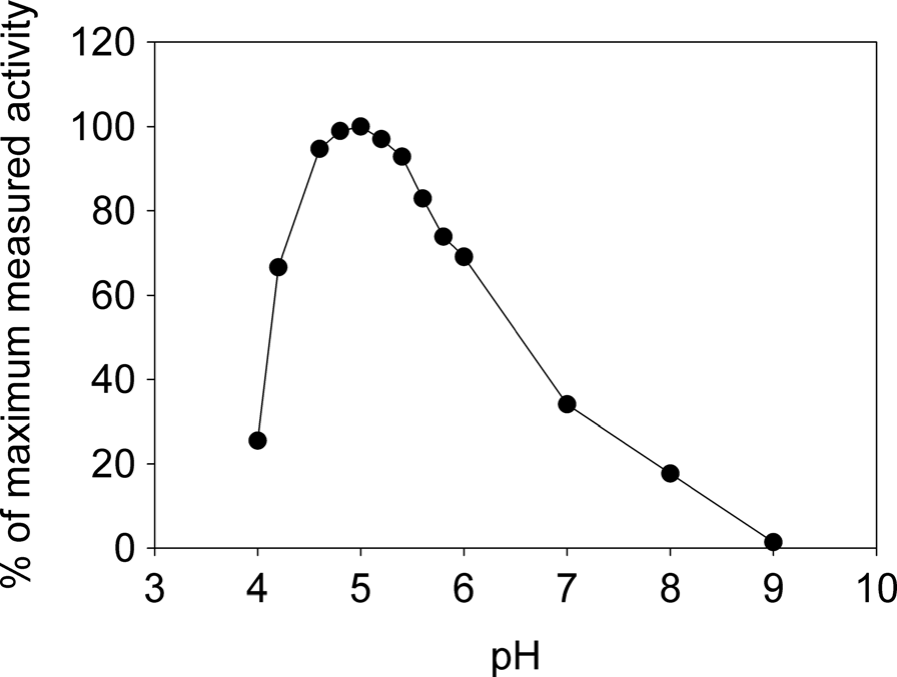
**Activity of recombinant *T. vaginalis *β-fructofuranosidase as a function of pH.** Purified β-fructofuranosidase (~27 nM, final concentration) was incubated at the indicated pH in the presence of sucrose and the initial rate of sucrose hydrolysis was determined. The maximum rate of hydrolysis was achieved at pH 5.0. To achieve a pH between 4.0 and 5.8, 20 mM sodium acetate buffer was used; for a pH between 6.0 and 8.0, 20 mM sodium phosphate buffer was employed; and pH 9.0 was reached using 20 mM Tris buffer. All buffers were supplemented with 50 mM NaCl. The results shown are the mean of two independent determinations, each performed in duplicate.

A survey of properties of various β-fructofuranosidases found in the BRENDA database revealed that the majority of such enzymes are most active below pH 7.0 [30,31]. Our preliminary characterization of the enzyme encoded by TVAG_254130 showed it to have greatest activity toward sucrose at acidic pH, with maximum activity being recorded from pH 4.8 to pH 5.2 (Figure 4). Less than 20% maximum activity remained at pH 8.0 and activity was barely detectable at pH 9.0. In temperature stability trials, we established that the enzyme lost only 25% of its activity towards sucrose after 30 min incubation at 50°C but that greater than 98% activity was lost after only 2 min incubation at 65°C (not shown). For practical reasons, we selected a temperature of 30°C and a pH of 5.0 as our standard assay conditions.

### Kinetic properties of the *T. vaginalis* β-fructofuranosidase

**Figure 5:**
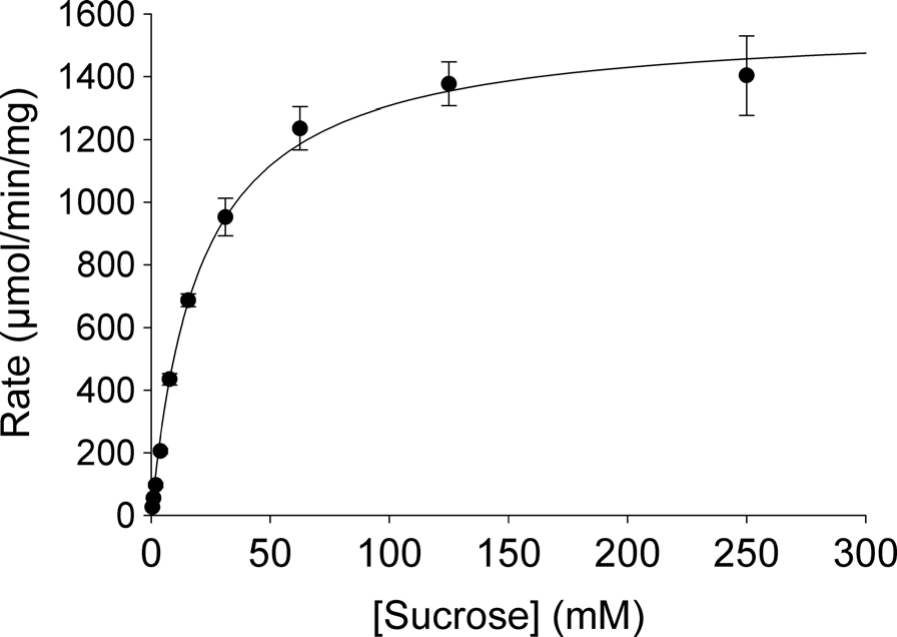
**Hydrolysis of sucrose by *T. vaginalis *β-fructofuranosidase.** Purified β-fructofuranosidase (~27 nM, final concentration) was incubated under standard assay conditions with the indicated concentrations of sucrose and the initial rate of sucrose hydrolysis was measured. Data were fitted to the Michaelis-Menten equation in order to determine kinetic constants. The results shown are the mean ± standard error for three independent experiments. K_m_ = 20.6 ± 2.2 mM; V_max_ = 1576 ± 48 μmol/min/mg.

To estimate the K_m_ and V_max_ for sucrose, we measured the initial rate of sucrose hydrolysis at different sucrose concentrations. The resulting data were fitted directly to the Michaelis-Menten equation as described in the ‘Methods’ section (Figure 5). The calculated K_m_ of 20.6 ± 2.2 mM and the V_max_ of 1576 ± 48 μmol/min/mg are both well within the range reported for purified β-fructofuronosidases from other species [30]. Indeed, the K_m_ for sucrose is comparable to that reported for the well-studied β-fructofuranosidase from budding yeast [32].

Having determined that the *T. vaginalis* β-fructofuranosidase had activity towards sucrose, we wanted to establish if other carbohydrates that contained terminal non-reducing fructose residues would serve as substrates. To these ends, we incubated the enzyme with raffinose (a trisaccharide of galactose, glucose, and fructose) or inulin (a polymer of fructose), both compounds that have been shown to serve as substrates for other characterized β-fructofuranosidases [31]. We established that the enzyme from *T. vaginalis* could use raffinose but that there was no detectable activity towards inulin (Table 2; not shown). Note that while the K_m_ for raffinose was around 3.7-fold lower than that for sucrose, the V_max_ was also reduced and the turnover number calculated for sucrose was some 4-fold higher than that for raffinose.

**Table 2:** **Kinetic properties of recombinant *T. vaginalis *** **β-fructofuranosidase**

**Substrate**	**K**_**m **_**(mM)**	**V**_**max **_**(μmol/min/mg)**	**Turnover number (s**^**-1**^**)**^**†**^
Sucrose	20.6 ± 2.2	1576 ± 48	1156 ± 36
Raffinose	5.5 ± 0.9	368 ± 16	270 ± 12

^†^Based upon a calculated molecular weight of 44 kDa for the recombinant protein.

Purified β-fructofuranosidase (~27 nM, final concentration) was incubated under standard assay conditions with the indicated concentrations of sucrose or raffinose and the initial rate of sugar hydrolysis was measured. Kinetic parameters were determined as described under ‘Methods’. For each substrate, the results shown are the mean ± standard error for three independent experiments.

## Discussion

We have established that extracts prepared from *T. vaginalis* cells grown under standard laboratory conditions with maltose as a carbon source have some capacity to hydrolyze sucrose, liberating glucose and fructose. Furthermore, we determined that the open reading frame TVAG_254130, predicted to encode a β-fructofuranosidase activity, is expressed under these same growth conditions. Lastly, when expressed in *E. coli*, the TVAG_254130 open reading frame yields a β-fructofuranosidase activity with robust activity towards sucrose and raffinose. Thus, it would appear reasonable to propose that the β-fructofuranosidase encoded by TVAG_254130 may well be responsible for the sucrose-degrading activity observed in the cell lysates. This degradative capacity notwithstanding, sucrose did not support the growth of *T. vaginalis*.

What accounts for the lack of growth on sucrose? To address this question, first consider how sucrose is used by other organisms. In the mammalian digestive system, sucrose is hydrolyzed to glucose and fructose by sucrase-isomaltase, an integral membrane protein preferentially expressed by enterocytes of the intestinal brush border membrane [33,34]. The glucose and fructose released are then transported into the enterocyte via specific carrier proteins for subsequent metabolism. Similarly, in the budding yeast *Saccharomyces cerevisae*, β-fructofuranosidase activity is secreted into the periplasmic space and sucrose is hydrolyzed there, the resulting monosaccharides being transported into the cell [32]. In other organisms, including many bacteria (see [35-38] for selected examples), plants (reviewed in [39]), some plant pathogenic fungi [40], and perhaps some insects [41], sucrose is transported into the cell by means of sucrose transport systems and then cleaved by internal β-fructofuranosidase activities. Although we detected β-fructofuranosidase activity associated with extracts of *T. vaginalis*, we were unable to determine any activity associated with growth medium in which *T. vaginalis* had been grown for 40 hr. While this observation does not allow us to formally exclude the possibility that some β-fructofuranosidase activity was secreted by the cells, we believe that this is likely not the case. Thus, we think it most probable that in order for *T. vaginalis* to utilize sucrose, it would first be transported into the cell.

The *T. vaginalis* genome sequence indicates coding capacity for 11 proteins that, based upon sequence similarity, are proposed to be sucrose transporters [18]. These proteins resemble known sucrose transporters from a variety of plants, as well as putative sucrose transporters from protists, such as *Dictyostelium sp.*, and bacteria. There is evidence from mass spectroscopy of *T. vaginalis*-derived peptides that at least three of these open reading frames are expressed (Mass Spec of T.vaginalis peptides from Richard Hayes, Patricia Johnson laboratory (version: 2009-05-27) accessed via the TrichDB database [19,21]). However, there is no direct experimental evidence that any of the putative sucrose transporter functions as such.

Regardless of the functionality or otherwise of these putative proteins in sucrose uptake, the presence of mRNA for TVAG_254130 in maltose-grown *T. vaginalis* and measurable β-fructofuranosidase activity in extracts from these same cells leads us to hypothesize that sucrose does not support growth of *T. vaginalis* under the conditions tested because it fails to enter the cells. This does not, of course, indicate that sucrose can never be used by *T. vaginalis*, only that sucrose was not utilized under the growth conditions that we tested.

## Conclusions

The natural habitat of *T. vaginalis* is the urogenital tract of both males and females and the most robust growth and proliferation occurs in the vagina [2]. Vaginal fluid contains a wealth of nutrients, including free amino acids, lipids, glucose, and other carbohydrates [42]. However, it is unclear where the organism would encounter sucrose or other fructose-containing compounds. It is interesting to note here that *T. vaginalis* is somewhat unusual in being unable to catabolize sucrose as several related organisms grow well with this carbon source. For example *P. hominis*, an inhabitant of the human large intestine, can grow using sucrose as a carbon source ([29]; our unpublished observations). Similarly *T. foetus*, best studied as a pathogen of the urogenital tract of cattle but now recognized as an important cause of feline diarrhea, also grows using sucrose [43-45]. Lastly, two trichomonads that infect the gastrointestinal tract of birds, *T. gallinae* and *T. gallinarum* can also use sucrose [12,46]. All of these organisms inhabit or can inhabit a niche where sucrose, raffinose, or other plant-derived oligosaccharides containing fructose might be found. It is likely that the most recent common ancestor of *T. vaginalis* was a gut-dwelling protist, where the capacity to utilize fructose-containing compounds might be advantageous [18]. Thus, *T. vaginalis* may retain the coding capacity and/or enzymatic activity to catabolize material that was abundant in an ancestral environment. The apparent drive towards increased genome size and increased cell size in *T. vaginalis* compared to related trichomonads [18] would mean that there was no pressure to lose such coding capacity, leading to the persistence of these ancestral sequences.

## Competing interest

The authors declare that they have no competing interests.

## Authors’ contributions

WAW and AB designed the research and wrote the manuscript. MD, MPB, and PP performed the research, analyzed the data, and assisted in writing the manuscript. All authors read and approved the final manuscript.
